# Supplementary material for: Evolution of PqsE as a Pseudomonas aeruginosa-specific regulator of LuxR-type receptors: insights from Pseudomonas and Burkholderia
Source: mBio. 2025 Apr 8;16(5):e00646-25. doi: 10.1128/mbio.00646-25 (PMC12077149; doi:10.1128/mbio.00646-25)

SUPPORTING INFORMATION

Table S1. NCBI BlastP results for PqsE homologs obtained from the *Pseudomonas* Genome DB[1]. Included in this table are standard BlastP output with *P. aeruginosa* PqsE used as a reference.

Table S2. Structural and sequence alignment table. This table shows the root mean square deviations and TM-score of predicted structures from *P. fluorescens* NCTC 10783, *B. cepacia* ATCC 25416, *B. thailandensis* E264, *B. pseudomallei* K96243 compared to the experimental structure of PqsE from *P. aeruginosa* (PDB ID: 5HIO[2]). This table also includes the percent amino acid identity and protein sequence length of each protein.

Table S3. Strains and plasmids used in this study. This table includes the names, genotypes, antibiotic resistance markers, and sources of all strains used in this study.

Table S4. Oligonucleotides used in this study. This table includes the names, sequences (5' to 3'), and description of each oligonucleotide used in this study.

Supplementary FIG 1. Sequence and structural comparison of PqsE and HhqE orthologs. (a) Multiple-sequence alignment of full-length protein sequences of PqsE from *P. aeruginosa* PA14 and *P. fluorescens* NCTC 10783 or HhqE in *B. cepacia* ATCC 25416, *B. pseudomallei* K96243, and *B. thailandensis* E264. Active site residues H69, H71, D73, and H74 (numbering according to PA14 PqsE) are highlighted in red. Residues involved in PqsE dimerization are highlighted in blue; residues unique to PqsE that are required for the PqsE-RhlR interaction are highlighted in green. Secondary structure assignments are represented above the sequence, determined from the PA14 PqsE experimental structure (PDB: 7KGW[3]). (b) Structural overlay of PA14 PqsE (PDB: 7KGW[3]) with predicted structures of the *Pseudomonas* and *Burkholderia* orthologs. For clarity, structures are depicted as ribbons, with the color scheme corresponding to the alignment in (a). All structure images were generated in ChimeraX[4-6].

Supplementary FIG 2. Estimation of PqsE, PqsE^{NI} and HhqE^{Bc} molecular weights by size exclusion chromatography. The peak elution profiles for PqsE, PqsE^{NI} and HhqE^{Bc} proteins were compared to peak elution volumes of MW protein standards: Vitamin B12 (1.35 kDa, V_e = 21.68 mL), myoglobin (17 kDa, V_e = 20.50 mL), ovalbumin (44 kDa, V_e = 16.49 mL), γ-globulin (158 kDa, V_e = 12.68), and thyroglobulin (670 kDa, V_e = 9.01). V_e indicates the peak elution volume. PqsE, PqsE^{NI} and HhqE^{Bc} had a V_e of 14.30 mL, 16.10 mL, and 16.45 mL respectively. The void volume (V_o) was determined at 8.08 mL as the elution volume of thyroglobulin. A standard curve was generated from a linear fit of the $\log_{10}$ MW (Da) of the protein standards versus the elution parameter K_{av} where $K_{av} = (V_e-V_o)/(V_c-V_o)$. V_c is equal to the total column volume. The MWs of PqsE, PqsE^{NI}, and HhqE^{Bc} were estimated to be 79 kDa, 34 kDa, and 32 kDa based on a linear fit to the MW protein standards.

Supplementary FIG 3. MassFludix HC experiments of PqsE, PqsE^{NI}, and HhqE^{Bc}. Mass photometry measurements of PqsE (A), PqsE^{NI} (B), and HhqE^{Bc} (C) following rapid dilution from 50 uM to 50 nM using the MassFluidix HC system (Refeyn). The number of counts is shown as a function of molecular weight (kDa). Mirror peaks in panels (B) and (C) arise due to the instrument's detection limit (50 kDa), reflecting PqsE^{NI}, and HhqE^{Bc}, the presence of which are too small to be directly measured.

Supplementary FIG 4. Structural similarities of LuxR-type receptors. Structural overlay of AlphaFold-Multimer predicted structures of CepR from *B. cepacia* and PmlR from *B. pseudomallei* compared to the experimental structure of RhlR (PDB ID: 8DQ0[7]). The percentage amino acid identity and root mean square deviation (rmsd) values of CepR and PmlR relative to PA14 RhlR are indicated. The inset highlights the universally conserved YXXXW motif within the ligand binding pocket (LBP) of LuxR-type receptors (numbering based on PA14 RhlR). The solvent-accessible volume of the RhlR LBP, shown in light blue is included to provide structural context. All structure images were generated in ChimeraX.

Supplementary FIG 5. HhqE does not complement pyocyanin production in a Δ*pqsE* strain of *P. aeruginosa*. (a) Pyocyanin production in Δ*pqsE* strains of *P. aeruginosa* expressing plasmid-borne *pqsE* or $hhqE^{Bc}$ was quantified by OD_{695}/OD_{600} and LC-MS. OD_{695}/OD_{600} values were normalized to levels observed in the strain expressing WT *pqsE* (center bar). (b) Growth of Δ*pqsE* strains carrying plasmids as in (a) on LB agar. Complementation of pyocyanin production was observed with WT *pqsE* but not with *hhqE* from *B. cepacia*.

Supplementary FIG 6. HhqE does not enhance CepR binding to promoter DNA. (a) EMSA analysis of *cepI* promoter DNA alone (minus symbol, left lane), with increasing concentrations of purified CepR:C_8HSL (left half), and with both CepR and HhqE (right half). An EMSA using *rhlA* promoter DNA was included as a negative control (middle). A representative SDS-PAGE gel shows protein levels for each sample (bottom). Final concentrations of CepR and HhqE were: 0 nm, 280 nM, 420 nM, 630 nM, 950 nM, and 1,350 nM.

REFERENCES

1. Winsor, G.L., Lam, D.K., Fleming, L., Lo, R., Whiteside, M.D., Yu, N.Y., Hancock, R.E. and Brinkman, F.S., 2010. *Pseudomonas* Genome Database: improved comparative analysis and population genomics capability for *Pseudomonas* genomes. *Nucleic acids research*, 39.suppl_1:D596-D600. https://doi.org/10.1093/nar/gkq869.

2. Taylor, I.R., Paczkowski, J.E., Jeffrey, P.D., Henke, B.R., Smith, C.D. and Bassler, B.L., 2021. Inhibitor mimetic mutations in the *Pseudomonas aeruginosa* PqsE enzyme reveal a protein–protein interaction with the quorum-sensing receptor RhlR that is vital for virulence factor production. *ACS chemical biology*, 16.4:740-752. https://doi.org/10.1021/acschembio.1c00049.

3. Zender, M., Witzgall, F., Drees, S.L., Weidel, E., Maurer, C.K., Fetzner, S., Blankenfeldt, W., Empting, M. and Hartmann, R.W., 2016. Dissecting the multiple roles of PqsE in *Pseudomonas aeruginosa* virulence by discovery of small tool compounds. *ACS chemical biology*, 11.6:1755-1763. https://doi.org/10.1021/acschembio.6b00156.

4. Meng, E.C., Goddard, T.D., Pettersen, E.F., Couch, G.S., Pearson, Z.J., Morris, J.H. and Ferrin, T.E., 2023. UCSF ChimeraX: Tools for structure building and analysis. *Protein Science*, 32.11:e4792. https://doi.org/10.1002/pro.4792.

5. Pettersen, E.F., Goddard, T.D., Huang, C.C., Meng, E.C., Couch, G.S., Croll, T.I., Morris, J.H. and Ferrin, T.E., 2021. UCSF ChimeraX: Structure visualization for researchers, educators, and developers. *Protein science*, 30.1:70-82. https://doi.org/10.1002/pro.3943.

6. Goddard, T.D., Huang, C.C., Meng, E.C., Pettersen, E.F., Couch, G.S., Morris, J.H. and Ferrin, T.E., 2018. UCSF ChimeraX: Meeting modern challenges in visualization and analysis. *Protein science*, 27.1:14-25. https://doi.org/10.1002/pro.3235.

7. Feathers, J.R., Richael, E.K., Simanek, K.A., Fromme, J.C. and Paczkowski, J.E., 2022. Structure of the RhlR-PqsE complex from *Pseudomonas aeruginosa* reveals mechanistic insights into quorum-sensing gene regulation. *Structure*, 30.12:1626-1636. https://doi.org/10.1016/j.str.2022.10.008.

8. Mukherjee, S., Moustafa, D.A., Stergioula, V., Smith, C.D., Goldberg, J.B. and Bassler, B.L., 2018. The PqsE and RhlR proteins are an autoinducer synthase–receptor pair that control virulence and biofilm development in *Pseudomonas aeruginosa*. *Proceedings of the National Academy of Sciences*, 115.40:E9411-E9418. https://doi.org/10.1073/pnas.1814023115.

9. McCready, A.R., Paczkowski, J.E., Henke, B.R. and Bassler, B.L., 2019. Structural determinants driving homoserine lactone ligand selection in the *Pseudomonas aeruginosa* LasR quorum-sensing receptor. *Proceedings of the National Academy of Sciences*, 116.1:245-254. https://doi.org/10.1073/pnas.1817239116.

10. Paczkowski, J.E., Mukherjee, S., McCready, A.R., Cong, J.P., Aquino, C.J., Kim, H., Henke, B.R., Smith, C.D. and Bassler, B.L., 2017. Flavonoids suppress *Pseudomonas aeruginosa* virulence through allosteric inhibition of quorum-sensing receptors. *Journal of Biological Chemistry*, 292.10:4064-4076. https://doi.org/10.1074/jbc.M116.770552.

11. Simanek, K.A., Taylor, I.R., Richael, E.K., Lasek-Nesselquist, E., Bassler, B.L. and Paczkowski, J.E., 2022. The PqsE-RhlR interaction regulates RhlR DNA binding to control

virulence factor production in *Pseudomonas aeruginosa*. *Microbiology spectrum*, 10.1:e02108-21. https://doi.org/10.1128/spectrum.02108-21.

Figure S1

A

```
                                β1         β2         β3             α1
P. aeruginosa      MLRLSAPGQLDDDLCLLGDVQVPVFLLRLGEASWALVEGGISRDAELVWADLCRWVAD--   58
P. fluorescens     MLRLSAPGQLDDDLCLLGDVQVPVFLLRLGEASWALVEGGISRDAELVWADLCRWVAD--   58
B. cepacia         -MLNASPGWIEPRLALIGTADVPLYVIVNDE-AATLIEGGLSGMTALVWQQLHDLLRDFG   58
B. pseudomallei    -MQHGTPGRIDSRLAIVGTADVPLYVIVNDE-AATLVEGGLSGMTELVWRQLHELLSDYG   58
B. thailandensis   -MQHSTPGWIDSQLAIVGTADVPLYVVVNDE-AATLVEGGLSGMTGLVWRQLHDLLKDYG   58

                      β4             α2                β5        α3           α4
P. aeruginosa      -PSQVHYWLITHKHYDHCGLLPYLCPRLPNVQVLASERTCQAWKSESAVRVVERLNRQLL   117
P. fluorescens     -PSQVHYWLITHKHYDHCGLLPYLCPRLPNVQVLASERTCQAWKSESAVRVVERLNRQLL   117
B. cepacia         GIRHLRYWLITHSHYDHCSLLMTLKSRMPWLHVSGSPDAFDAFQSPSACRTIRQLDEHAS   118
B. pseudomallei    GIRHLRYWLITHSHYDHCSLLGTLAPRMPWLHIVGSAATADALQSPSACRTIRALDAQAC   118
B. thailandensis   GIRHLRYWLITHSHYDHCSLLGTLAPRMPWLHIVGSPETADALQSPSARRTIRKLDAQAS   118

                                          β6               β7           β8
P. aeruginosa      RAEQRLP--EACAWDALPVRAVADGEWLELGPRHRLQVIEAHGHSDDHVVFYDVRRRRLF   175
P. fluorescens     RAEQRLP--EACAWDALPVRAVADGEWLELGPRHRLQVIEAHGHSDDHVVFYDVRRRRLF   175
B. cepacia         RSWEPAAGADFTELSDLPFYPVNPGAQLDIGDGMQIRTIALPGHSRCQFGYYCPQLDIGF   178
B. pseudomallei    AAWEPVAVA--AELSDIPLYPLNPGRSLDIGEGMRMRALALPGHSACLLGYHCPQLDLLF   176
B. thailandensis   DAWEPVADADLADLSDIPLYPLNPGRALDIGEGMRMRAVALPGHSACLLGYHCPQLDLLF   178

                      β9                     α5              β10
P. aeruginosa      CGDALGEFDEAEGVWRPLVFDDMEAYLESLERLQRLPTLLQLIPGHGGLLRGRLAADGAE   235
P. fluorescens     CGDALGEFDEAEGVWRPLVFDDMEAYLESLERLQRLPTLLQLIPGHGGLLRGRLAADGAE   235
B. cepacia         VSDALGEFQDA-THWLPLVFQDLFAYRHSLDVIEQLHAP-RLALGHHGILTGELARSAAR   236
B. pseudomallei    VSDALGEYHAP-TQWLPLVFQDLAAYRLSLDAIERQHAS-RIALGHHGIVGGGIARHAVR   234
B. thailandensis   VSDALGEYHAP-TQWLPLVFQDLPAYRQSLDEIERRHAS-RIALGHHGIVGGDVARHAVR   236

                           α6                  α7                   α8
P. aeruginosa      SAYTECLRLCRRLLWRQSM--GESLDELSEELHRAWGGQSVDFLPGELHLGSMRRMLEIL   293
P. fluorescens     SAYTECLRLCRRLLWRQSM--GESLDELSEELHRAWGGQSVDFLPGELHLRSMRRMLEIL   293
B. cepacia         HAR-ACLDAREA-DARAVRGNAAATQALAHQWTARYAARSEKVVPRFLHLKSMTHMIDLF   294
B. pseudomallei    HAR-DGLAARDD-EARAARGDANATRALARQWTERYAARSANVVPRALHLKSMERMIDLF   292
B. thailandensis   HAR-DGLAARGD-EARAASGDANATRALAQQWTERYAARSEKVVPRALHLKSMERMIDLF   294

P. aeruginosa      SRQALPLD   301
P. fluorescens     SRQALPLD   301
B. cepacia         HRAE----   298
B. pseudomallei    QRAA----   296
B. thailandensis   QRAA----   298
```

B

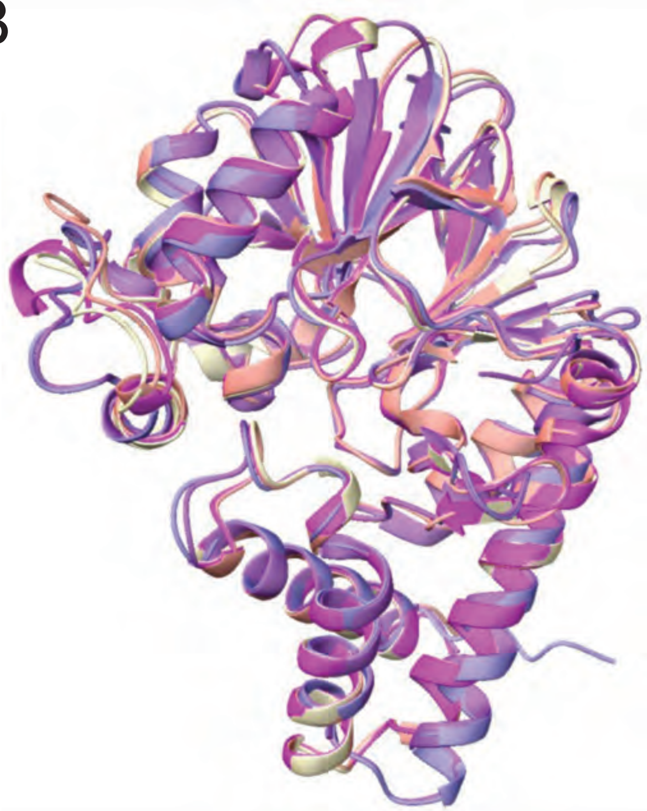

Figure S2

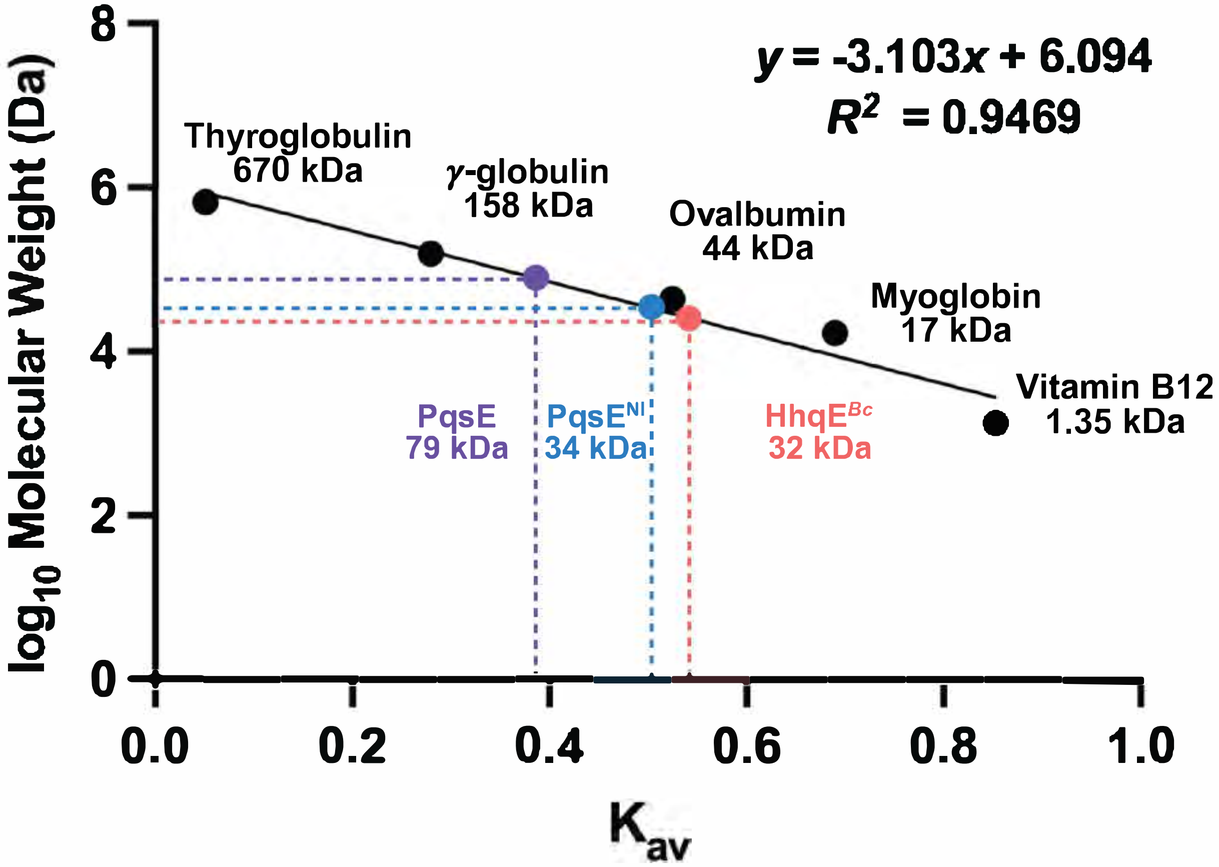

y = -3.103x + 6.094
R2 = 0.9469
Thyroglobulin
670 kDa
γ-globulin
158 kDa
Ovalbumin
44 kDa
Myoglobin
17 kDa
Vitamin B12
1.35 kDa
PqsE
79 kDa
PqsENI
34 kDa
HhqEBc
32 kDa
log10 Molecular Weight (Da)
Kav
0
2
4
6
8
0.0
0.2
0.4
0.6
0.8
1.0

Figure S3

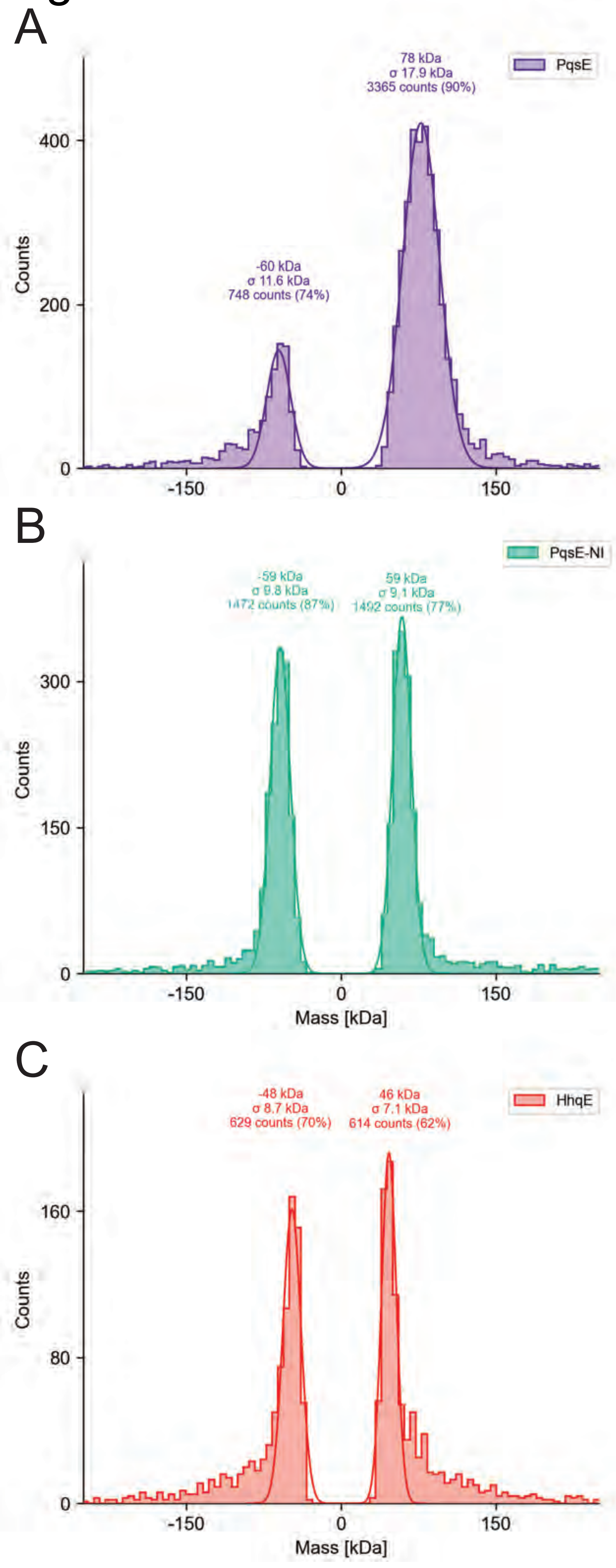
A
PqsE
78 kDa
σ 17.9 kDa
3365 counts (90%)
-60 kDa
σ 11.6 kDa
748 counts (74%)
Counts
B
PqsE-NI
-59 kDa
σ 9.8 kDa
1472 counts (87%)
59 kDa
σ 9.1 kDa
1492 counts (77%)
Counts
Mass [kDa]
C
HhqE
-48 kDa
σ 8.7 kDa
629 counts (70%)
46 kDa
σ 7.1 kDa
614 counts (62%)
Counts
Mass [kDa]

Figure S4

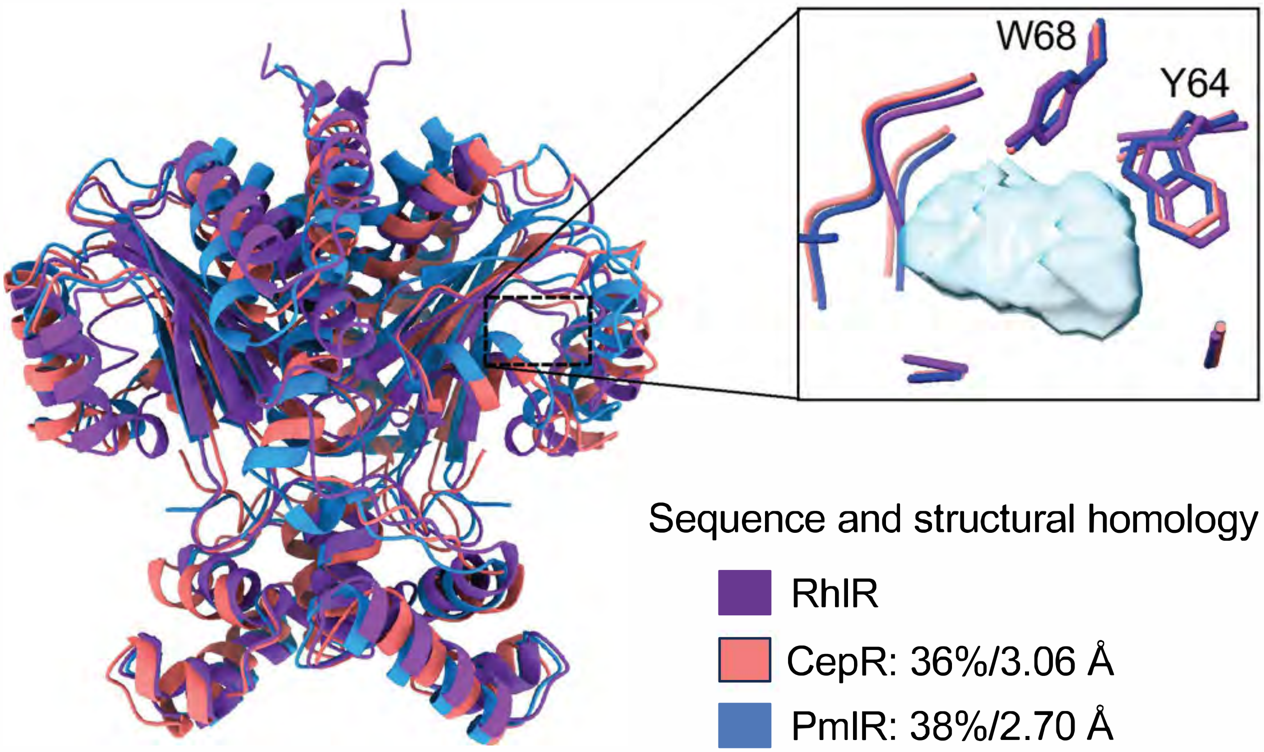

W68
Y64
Sequence and structural homology
RhlR
CepR: 36%/3.06 Å
PmlR: 38%/2.70 Å

Figure S5

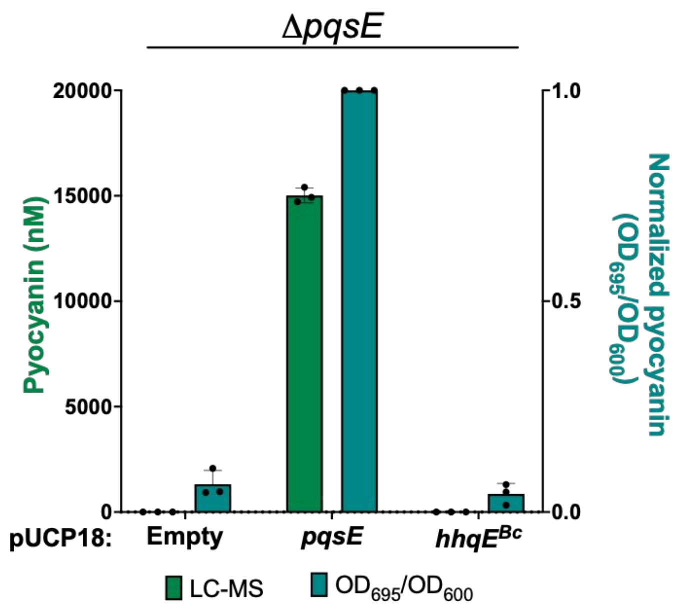

ΔpqsE
Pyocyanin (nM)
Normalized pyocyanin (OD695/OD600)
20000
15000
10000
5000
0
1.0
0.5
0.0
pUCP18:
Empty
pqsE
hhqE^Bc
LC-MS
OD695/OD600

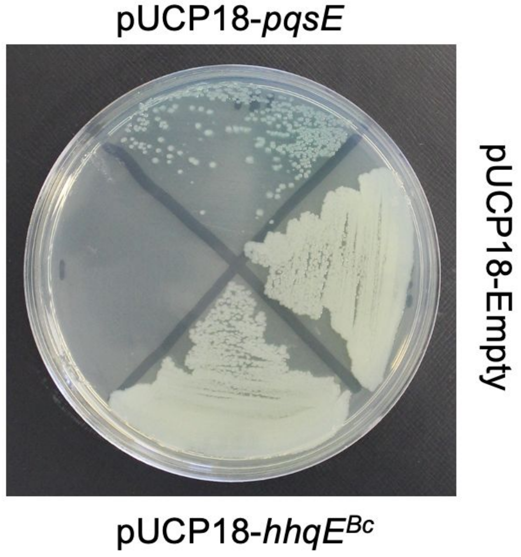

pUCP18-pqsE
pUCP18-Empty
pUCP18-hhqE^Bc

Figure S6

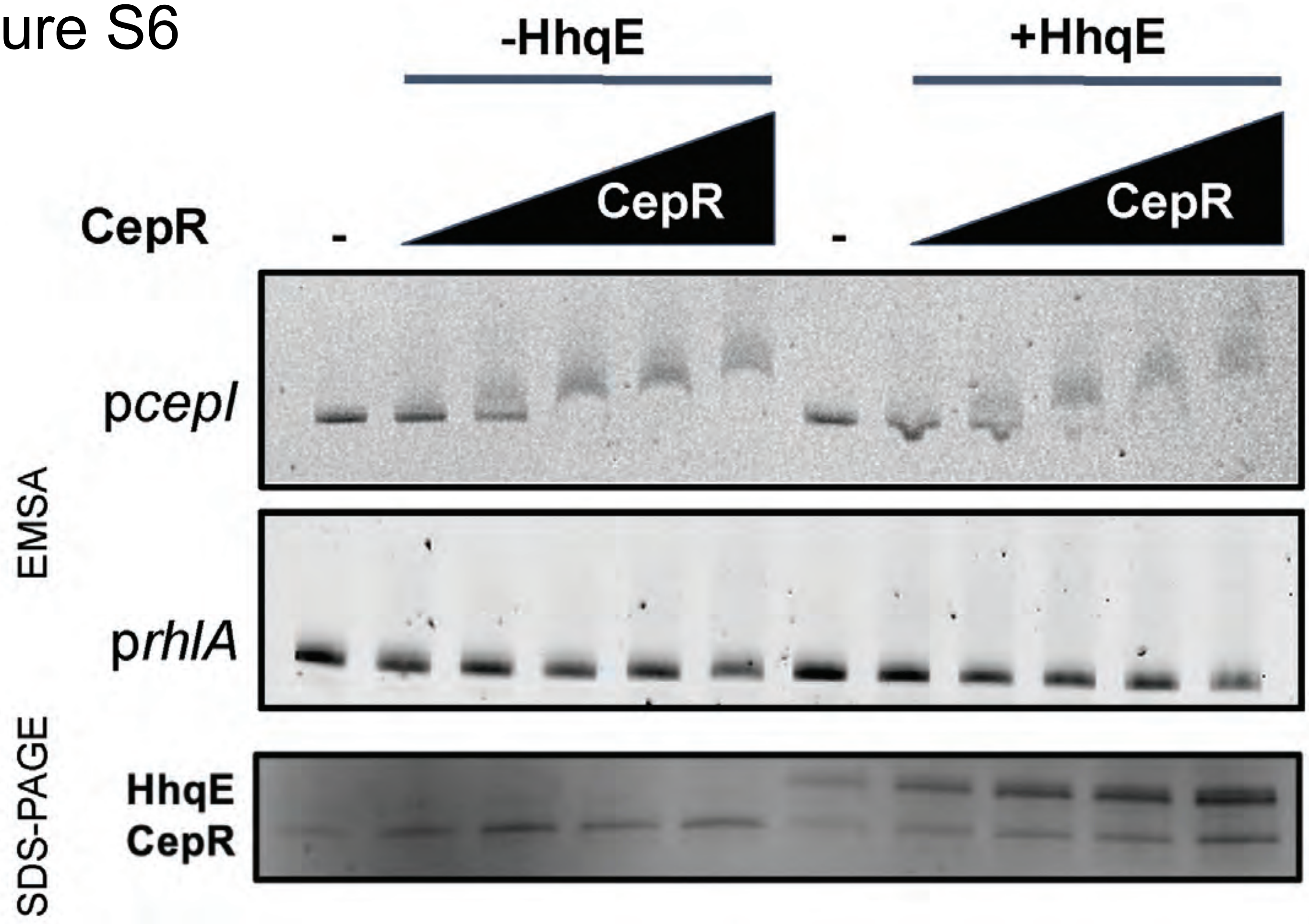

Supplement: Supplemental Material — Supplemental figures, table captions, and references. [file mbio.00646-25-s0001.pdf]
