# Supplementary figures and images for: Evolution of PqsE as a Pseudomonas aeruginosa-specific regulator of LuxR-type receptors: insights from Pseudomonas and Burkholderia
Source: mBio. 2025 Apr 8;16(5):e00646-25. doi: 10.1128/mbio.00646-25 (PMC12077149; doi:10.1128/mbio.00646-25)

## Table S1

[illegible]

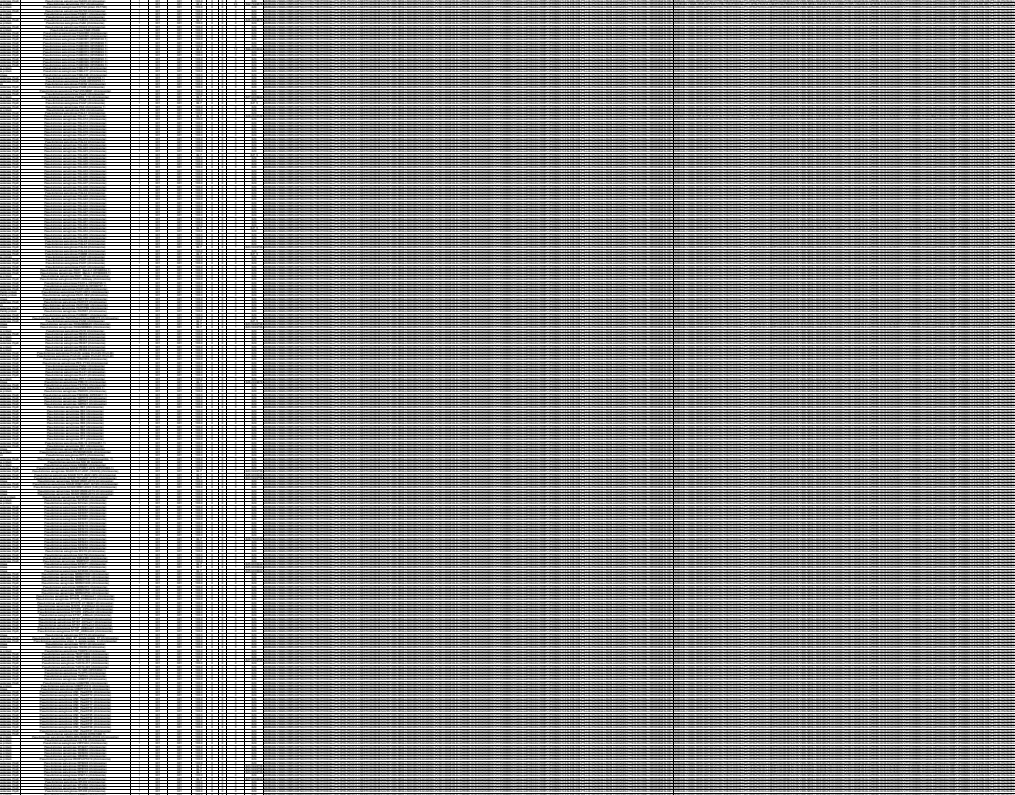

Supplement: Table S1 — NCBI BlastP results for PqsE homologs obtained from the Pseudomonas Genome DB. [file mbio.00646-25-s0002.pdf]
