# Supplementary material for: Evolution of PqsE as a Pseudomonas aeruginosa-specific regulator of LuxR-type receptors: insights from Pseudomonas and Burkholderia
Source: mBio. 2025 Apr 8;16(5):e00646-25. doi: 10.1128/mbio.00646-25 (PMC12077149; doi:10.1128/mbio.00646-25)
Supplement: Table S2 — Structural and sequence alignment table. [file mbio.00646-25-s0003.pdf]

Table S2

PDB/ID	RMSD	TM-score	Identity	Sequence length
5HIO	-	-	-	301
P. fluorescens PqsE	0.47	0.99	99%	301
B. cepacia HhqE	1.80	0.92	31%	298
B. pseudomallei HhqE	1.90	0.92	30%	298
B. thailandensis HhqE	1.91	0.92	30%	298
