# Supplementary material for: Evolution of PqsE as a Pseudomonas aeruginosa-specific regulator of LuxR-type receptors: insights from Pseudomonas and Burkholderia
Source: mBio. 2025 Apr 8;16(5):e00646-25. doi: 10.1128/mbio.00646-25 (PMC12077149; doi:10.1128/mbio.00646-25)
Supplement: Table S3 — Strains and plasmids used in this study. [file mbio.00646-25-s0004.pdf]

Table S3

Strain name	Genotype	Plasmid	Antibiotic resistance	Source
JPS0102	*E. coli* BL21	pET28-*pqsE*	Kan	Mukherjee *et al.* (2018)
JPS0103	*E. coli* BL21	pET23-*rhlR*	Amp	McCready *et al.* (2019)
JPS0118	*E. coli* TOP10	pBAD-A-*rhlR* pCS26-p*rhlA*-*luxCDABE*	Amp/Kan	Paczkowski *et al.* (2017)
JPS0151	*P. aeruginosa* Δ*rhlR*			Mukherjee *et al.* (2018)
JPS0154	*P. aeruginosa* Δ*rhlI*			Mukherjee *et al.* (2018)
JPS0222	*P. aeruginosa* (UCBPP-PA14) WT			Gift from Dr. George O'Toole
JPS0225	*E. coli* DH5a	pET-DUET empty	Amp	Invitrogen
JPS0226	*E. coli* DH5a	pBAD-A empty	Amp	Invitrogen
JPS0227	*E. coli* DH5a	pEXG2 empty	Gent	Gift from Dr. Joseph Mougous
JPS0247	*E. coli* Top 10	pUCP18 empty	Amp	Mukherjee *et al.* (2018)
JPS0268	*P. aeruginosa* Δ*pqsE*	pUCP18-empty	Carb	Simanek *et al.* (2022)
JPS0278	*P. aeruginosa* Δ*rhlI* Δ*pqsE*			Simanek *et al.* (2022)
JPS0375	WT *Burkholderia cepacia*			ATCC
JPS0544	*P. aeruginosa* PqsE (R243A/R246A/R247A)			Simanek *et al.* (2022)
JPS0561	*P. aeruginosa* Δ*pqsE*			Simanek *et al.* (2022)
JPS0592	*P. aeruginosa* Δ*rhlI* PqsE (R243A/R246A/R247A)			Simanek *et al.* (2022)
JPS0808	*E. coli* DH5a	pACYC-empty	Tet	Taylor *et al*. (2021)
JPS1067	*E. coli* DH5a	pET28-*hhqE* (*B. cepacia*)	Kan	This study
JPS1068	*E. coli* BL21	pET28-*hhqE* (*B. cepacia*)	Kan	This study
JPS1069	*E. coli* DH5a	pET-DUET-*cepR* (B. cepacia)	Amp	This study
JPS1070	*E. coli* BL21	pET-DUET-*cepR* (B. cepacia)	Amp	This study
JPS1223	*E. coli* DH5a	pET28-*pqsE* (*P. fluorescens* NCTC 10783)	Kan	This study
JPS1224	*E. coli* DH5a	pET28-*hhqE* (*B. pseudomallei*)	Kan	This study
JPS1225	*E. coli* DH5a	pET28-*pqsE* (*P. fluorescens* NCTC 10783)	Kan	This study
JPS1228	*E. coli* DH5a	pET-DUET-*pmlR* (*B. pseudomallei*)	Amp	This study
JPS1233	*E. coli* BL21	pET28-*pqsE* (*P. fluorescens* NCTC 10783)	Kan	This study
JPS1234	*E. coli* BL21	pET-DUET-*pmlR* (*B. pseudomallei*)	Amp	This study
JPS1305	*E. coli* DH5a	pUCP18-*hhqE* (*B. cepacia)*	Amp	This study
JPS1308	*P. aeruginosa* Δ*pqsE*	pUCP18-*hhqE* (*B. cepacia)*	Carb	This study
JPS1511	*E. coli* DH5a	pBAD-A-*cepR* (*B. cepacia*)	Amp	This study
JPS1593	*E. coli* Top 10	pACYC-*hhqE* (*B.cepacia*)	Tet	This study
JPS1646	E. coli Top 10	pBAD-A-*cepR* pCS26-p*cepI*-*luxCDABE*	Amp/Kan	This study
JPS1647	E. coli Top 10	pBAD-A-*rhlR* pCS26-p*rhlA*-luxCDABE pACYC-*hhqE* (*B. cepacia*)	Amp/Kan/Tet	This study
JPS1663	*E. coli* Top 10	pBAD-A-empty pCS26-p*cepI*-*luxCDABE*	Amp/Kan	This study
JPS1715	*E. coli* Top 10	pACYC-*pqsE* (*P. fluorescens* NCTC 10783)	Tet	This study
JPS1732	*E. coli* Top 10	pBAD-A-*rhlR* pCS26-p*rhlA*-*luxCDABE* pACYC-*pqsE* (*P. fluorescens* NCTC 10783)	Amp/Kan/Tet	This study
JPS1737	*E. coli* DH5a	pUCP18-*pqsE* (*P. fluorescens* NCTC 10783)	Amp	This study
JPS1918	*E. coli* DH5a	pEXG2-Δ*lon*	Gent	This study
JPS1919	*E. coli* SM10-λpiR	pEXG2-Δ*lon*	Gent	This study
JPS1920	*P. aeruginosa* Δ*lon*			This study
JPS1921	*P. aeruginosa* Δ*pqsE* Δ*lon*			This study
