# Supplementary material for: Evolution of PqsE as a Pseudomonas aeruginosa-specific regulator of LuxR-type receptors: insights from Pseudomonas and Burkholderia
Source: mBio. 2025 Apr 8;16(5):e00646-25. doi: 10.1128/mbio.00646-25 (PMC12077149; doi:10.1128/mbio.00646-25)
Supplement: Table S4 — Oligonucleotides used in this study. [file mbio.00646-25-s0005.pdf]

Table S4

Primer name	Primer sequence	Description
JP0484	AGGCCTGCGAAGTGTCCTAT	*rhlA* promoter 300 bp (forward) EMSA probe
JP0485	TTCACACCTCCCAAAAATTT	*rhlA* promoter 300 bp (reverse) EMSA probe
JP1083	gatataccatgggcagcagc	pET28 (forward) for overlap extension
JP1084	tttcgggctttgttagcagc	pET28 (reverse) for overlap extension
JP1085	gcaggtaagctaattccaca	pEXG2 (forward) for overlap extension
JP1086	ggtaccgaattcgagctcga	pEXG2 (reverse) for overlap extension
JP1438	ttaa catatg atgctgaacgcatcccccgg	*hhqE* (*B. cepacia*) (forward) for pET28
JP1439	ttaa ctcgag ctattcggcgcgatggaaca	*hhqE* (*B. cepacia*) (reverse) for pET28
JP1444	ttaa CCATGG atggaactgcgctggcagga	*cepR* (*B. cepacia*) (forward) for pET-DUET
JP1445	ttaa AAGCTT tcagggcgcctcgatcagtc	*cepR* (*B. cepacia*) (reverse) for pET-DUET
JP1928	CTACAA CATATG CTGCGACTTTCTGCCCCAGGC	*pqsE* (*P. fluorescens* NCTC10783) (forward) for pET28
JP1929	CGCCA CTCGAG TTAATCGAGTGGCAAAGC	*pqsE* (*P. fluorescens* NCTC10783) (reverse) for pET28
JP1930	CTACAA CATATG GAACTGCGCTGGCAAGACG	*pmlR* (*B. pseudomallei*) (forward) for pET-DUET
JP1931	CGCCA GGTACC TTACGGCGCGTCGATGAGC	*pmlR* (*B. pseudomallei*) (reverse) for pET-DUET
JP1932	CTACAA CATATG CAACATGGAACCCCG	*hhqE* (*B. pseudomallei*) (forward) for pET28
JP1933	CGCCA CTCGAG TTATGCTGCTCTTTGAAAC	*hhqE* (*B. pseudomallei*) (reverse) for pET28
JP1987	ttaa AAGCTT atgctgaacgcatcccccgg	*hhqE* (*B. cepacia*) (forward) for pUCP18
JP1988	ttaa ggatcc ctattcggcgcgatggaaca	*hhqE* (*B. cepacia*) (reverse) for pUCP18
JP2219	cagctatgaccatgattacgATGCAACATGGAACCCCGG	*hhqE* (*B. pseudomallei*) (forward) for pUCP18 use w/JP2481
JP2081	gcaatggatgtcccgccTTATTCGGCGCGATGGAACAGGTCG	*hhqE* (*B. cepacia*) (forward) for pACYC
JP2082	GAATTCAGGAGGTGAACCATGCTGAACGCATCCCCCG	*hhqE* (*B. cepacia*) (reverse) for pACYC
JP2261	TACAGATTAAATCAGAACGC	pBAD-A (forward) for Hifi
JP2262	GGCTGAAAATCTTCTCTC	pBAD-A (reverse) for Hifi
JP2169	CGGCTCGCGTGCATCGACTGGC	*cepI* promoter 300 bp (forward) EMSA probe
JP2170	GGTGTCCTCGGATTTGTGC	*cepI* promoter 300 bp (reverse) EMSA probe
JP2171	gctaacaggaggaattaaccATGGAACTGCGCTGGCAG	*cepR* (*B. cepacia*) (forward) for pBAD-A
JP2172	ttctgatttaatctgtatcaTCAGGGCGCCTCGATCAG	*cepR* (*B. cepacia*) (reverse) for pBAD-A
JP2201	tcgtcttcacctcgaggggaCACGCCTCGCCGGTTATAC	*cepI* promoter 300 bp (forward) for pCS26
JP2202	atgatccacctcctggatccGGTGTCCTCGGATTTGTGC	*cepI* promoter 300 bp (reverse) for pCS26
JP2217	GGCACTGGCCGTCGTTTTAC	pUCP18 (forward) for Hifi
JP2293	cagctatgaccatgattacgATGTTGAGGCTTTCGGCTCC	*pqsE* (*P. fluorescens* NCTC10783) (forward) for pUCP18 use w/JP2482
JP2295	attcaggaggtgaaccggccATGTTGAGGCTTTCGGCTCC	*pqsE* (*P. fluorescens* NCTC10783) (forward) for pACYC
JP2296	gcaatggatgtcccgccttaTCAGTCCAGAGGCAGCGC	*pqsE* (*P. fluorescens* NCTC10783) (reverse) for pACYC
JP2436	gtaaagcaagcttctgcaggGTAGGGCGTTTGCCGGTG	*lon* 500 bp upstream (forward) for pEXG2
JP2437	tacctaccgaAATGTCGGCTCTACAGCGG	*lon* 500 bp upstream (reverse) for pEXG2
JP2438	agccgacattTCGGTAGGTATTCTTGACACTGTTTTTG	*lon* 500 bp downstream (forward) for pEXG2
JP2439	aattaattaaggtaccgaatGCGTGACAGGCCGGTATTC	*lon* 500 bp upstream (reverse) for pEXG2
JP2476	gtaatcatggtcatagctgtttcc	pUCP18 (reverse) for Hifi
JP2481	ggaaacagctatgaccatgattacTTATGCTGCTCTTTGA	*hhqE* (*B. pseudomallei*) (reverse) for pUCP18 use w/JP2219
JP2482	ggaaacagctatgaccatgattacTCAGTCCAGAGGCAGCGC	*pqsE* (*P. fluorescens* NCTC10783) (reverse) for pUCP18 use w/JP2293
